# A cardiovascular magnetic resonance (CMR) safe metal braided catheter design for interventional CMR at 1.5 T: freedom from radiofrequency induced heating and preserved mechanical performance

**DOI:** 10.1186/s12968-019-0526-7

**Published:** 2019-03-07

**Authors:** Korel D. Yildirim, Burcu Basar, A. E. Campbell-Washburn, Daniel A. Herzka, Ozgur Kocaturk, Robert J. Lederman

**Affiliations:** 1 Institute of Biomedical Engineering, Bogazici University, Istanbul, Turkey; 2 Cardiovascular Branch, Division of Intramural Research, National Heart Lung and Blood Institute, National Institutes of Health, Building 10, Room 2c713, Bethesda, MD 20892-1538 USA

**Keywords:** Interventional MRI, Cardiovascular magnetic resonance, CMR catheterization, Catheter engineering, Catheterization methods, Equipment design

## Abstract

**Background:**

Catheter designs incorporating metallic braiding have high torque control and kink resistance compared with unbraided alternatives. However, metallic segments longer than a quarter wavelength (~ 12 cm for 1.5 T scanner) are prone to radiofrequency (RF) induced heating during cardiovascular magnetic resonance (CMR) catheterization. We designed a braid-reinforced catheter with interrupted metallic segments to mitigate RF-induced heating yet retain expected mechanical properties for CMR catheterization.

**Methods:**

We constructed metal wire braided 6 Fr catheter shaft subassemblies using electrically insulated stainless-steel wires and off-the-shelf biocompatible polymers. The braiding was segmented, in-situ, using lasers to create non-resonant wire lengths. We compared the heating and mechanical performance of segmented- with un-segmented- metal braided catheter shaft subassemblies.

**Results:**

The braiding segmentation procedure did not significantly alter the structural integrity of catheter subassemblies, torque response, push-ability, or kink resistance compared with non-segmented controls. Segmentation shortened the electrical length of individually insulated metallic braids, and therefore inhibited resonance during CMR RF excitation. RF-induced heating was reduced below 2 °C under expected use conditions in vitro.

**Conclusion:**

We describe a simple modification to the manufacture of metallic braided catheters that will allow CMR catheterization without RF-induced heating under contemporary scanning conditions at 1.5 T. The proposed segmentation pattern largely preserves braid structure and mechanical integrity while interrupting electrical resonance. This inexpensive design may be applicable to both diagnostic and interventional catheters and will help to enable a range of interventional procedures using real time CMR.

## Background

Cardiovascular magnetic resonance (CMR) catheterization is an attractive strategy because it avoids ionizing radiation and affords superior soft tissue contrast in arbitrary imaging planes, in real-time, at speeds up to 10 frames per second [1–5]. Clinical adoption of CMR catheterization remains limited because clinical grade CMR-compatible devices, e.g. guidewires and catheters, are currently not available for safe and effective catheter procedures in patients [6–8]. Limited diagnostic and therapeutic CMR catheterization procedures have been embraced by a handful of clinical centers by using either CMR compatible commercially available devices or clinical grade CMR-conditional device prototypes or both [9, 10]. CMR catheter devices typically omit metallic components to reduce image distortion and enhance CMR compatibility and radiofrequency (RF) safety [11]. However, non-metallic components reduces the overall mechanical performance of the medical devices adversely [12, 13], and limits their suitability for interventional procedures.

Ideally, a CMR-compatible catheter must have a comparable mechanical performance to commercially available devices designed to perform a similar procedure under fluoroscopy. Commercial catheters incorporate metal braids or coils along the shaft to impart torque response, push-ability and kink resistance. However, such designs are intrinsically susceptible to radiofrequency (RF)-induced heating under CMR, risking thermal injury [13–16]. Replacing metal wires with polymer fibers in braiding layer avoids RF-induced heating, however results in inferior mechanical, hence operational, performance during CMR catheterization [12]. However, if the electrical length of each metal component used in catheter construction is restricted to be less than a quarter-wavelength at a specified electromagnetic field, standing wave formation thus RF-induced heating can be reduced or eliminated [14–16]. At 1.5 Tesla (T), a quarter wavelength within the body is approximately 12 cm [17]. In a previous work we demonstrated that CMR-safe guidewires can be constructed using by mechanically joining but electrically isolating quarter-wavelength metallic segments [18]. Here, we demonstrate an CMR safe full metal braided catheter design by segmenting each individual braid wire such a way that the electrical continuity is disrupted every 10 cm along the wire while maintaining circumferential braid continuity over the whole shaft. This allows standard commercial manufacturing procedures to be largely preserved in building CMR safe metallic braided catheters.

## Methods

We compared heating, in-vitro and in-vivo imaging and mechanical performance of conventional braided steel catheter shaft subassemblies with segmented variants described below. These used commercial clinical grade components assembled in our laboratory. A key difference between our subassemblies and standard commercial clinical subassemblies is that we used electrically insulated steel wires for construction, so crossed braiding wires would not create short-circuits. As there is no commercially available CMR compatible metal braided catheter, we compared mechanical properties of in-house developed prototypes with a commercially available but not CMR compatible metal braided catheter (6F Supertorque Angiographic Catheter, Cordis Cardinal Health, Miami Lakes, Florida, USA).

### Prototype construction

**Fig. 1:**
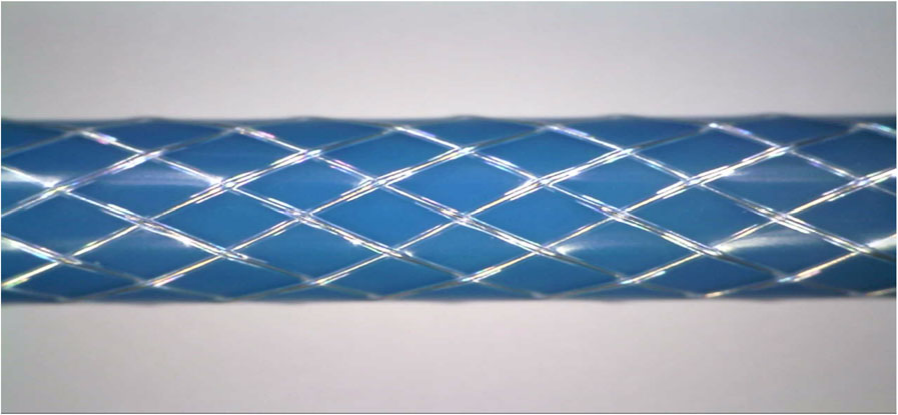
Embedded braiding layer between two Pebax layers after reflowing process

We first constructed a 6 French (Fr) stainless steel braided catheter prototype. A full load braid pattern (2-under-2-over or diamond pattern) as is often seen in most commercial braided catheter designs [19] was formed in a standard vertical braider (K80/16, Steeger Inc., Spartanburg County, South Carolina, USA) by using perfluoroalkoxyalkane (PFA)-insulated 316 L stainless steel wires (0.008″ diameter bare wire insulated with 0.001″ thick PFA polymer layer, A-M Systems, Sequim, Washington, USA). A polytetrafluoroethylene (PTFE) lined blue color thermoplastic polymer tubing with 0.080″ inner diameter (ID) and 0.096″ outer diameter (OD) (72D Pebax, Zeus Inc., Orangeburg, South Carolina, USA) was used as the inner layer of the catheter. Insulated stainless steel wires were braided over the inner Pebax layer using 10 pitch per inch (PPI) and 50 rpm table speed settings. Another clear Pebax polymer layer was applied on top of the assembly as the outer layer. Both Pebax layers were reflowed by heating (Model 210-A, Beahm Designs Inc., Milpitas, California, USA) so that the metal braiding layer was embedded between two layers (Fig. 1).

In our selected braiding pattern, a pair of interweaved wires completes its 360-degree rotation and interweaves again, after 9 nodes (spots where the wires cross). The linear length (LL) of a single wire after a 360-degree rotation can be calculated using the Pythagorean theorem (Eq. 1).

1 $$ LL=\sqrt{LD^2+{PC}^2} $$

where LD is the distance on horizontal axis after a 360-degree rotation and the PC is the perimeter of the catheter tube (braiding wire diameter is ignored). LD can be calculated using Eq. 2,

2 $$ LD=8\cdot NN $$

where NN is the horizontal node-to-node distance of the braiding.

**Fig. 2:**
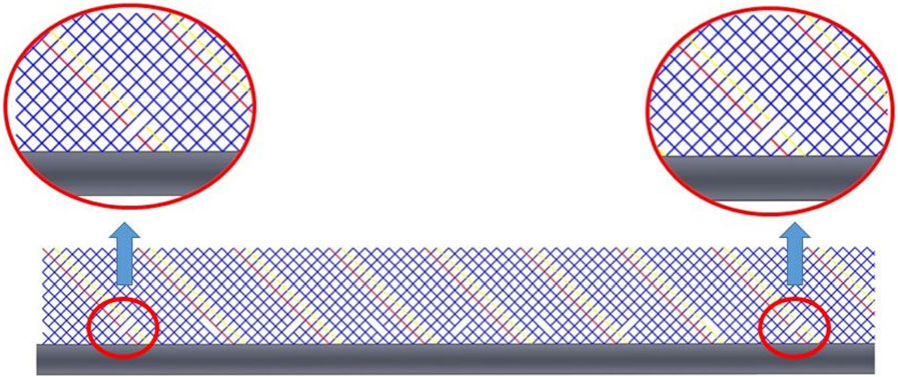
Schematic of a full-cycle braiding segmentation pattern. Breaks on the braid pattern can be seen by following the red and yellow lines. The pattern is shown in 2-D, representing the unwrapped form of the catheter shaft

NN was measured as 1.35 mm and linear length of the wire was calculated as 1.28 cm. Every braiding wire was cut in 10.24 cm intervals using a 1064 nm Nd:YAG laser system (Lasag Inc., Belp, Switzerland) to process all wires in less than a quarter wavelength and eliminate RF-induced heating (Fig. 2). At each segmentation point along the catheter, laser cutting was done on a pair of adjacent wires (two wires out of 16 wires each time) as shown in Fig. 2. By following this segmentation pattern, electrical continuity of each braiding wire was interrupted at less than a quarter wave-length while sustaining a continuous braid structure.

The biggest differences between the materials used in the catheters we designed and commercial braided catheters include the use of PFA polymer for insulation and the choice of 316 L stainless steel. PFA polymer was used for electrical isolation of crossing braiding wires, a must to prevent short circuits. We chose 316 L surgical stainless steel as the braiding wire because it creates a modest susceptibility artifact compared with the common alternative 304 stainless steel. Compared with current commercial braided catheters, we add a single additional manufacturing step consisting of in-situ laser segmentation.

### RF-induced heating

**Fig. 3:**
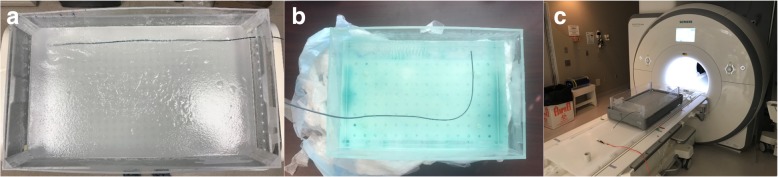
**a** Test sample position in the ASTM 2182 gel phantom, **b** in the ASTM 2119 copper sulfate phantom and **c** phantom and sample positions in the scanner

Segmented braided catheter prototypes (100 cm long) were compared with equivalent length but non-segmented braided catheter samples in terms of RF-induced heating performance. The segmented and non-segmented catheter prototypes were tested for RF-induced heating per ASTM standard 2182 [20] at 1.5 T (Aera, Siemens Healthineers, Erlangen, Germany) using a balanced steady-state free precession (bSSFP) (TE/TR = 1.05/283.63 ms, flip angle = 45°, FOV = 300 mm, matrix = 192 × 192, slice thickness = 10 mm) and a gradient echo (GRE) sequence (TE/TR = 2.97/727.72 ms, flip angle = 45°, FOV = 300 mm, matrix = 192 × 192, slice thickness = 10 mm) which are commonly used real-time interventional CMR sequences. Real-time bSSFP was chosen because it is the workhorse pulse sequence used in clinical CMR catheterization, and because it has relatively high average specific absorption rate (SAR) values [21, 22]. In addition, we tested high flip angle real-time GRE. A fiberoptic temperature probe (OTG M170, OpSens Inc. Quebec, Canada) was introduced into a thin-wall polyamide tube affixed to the outer surface of catheter samples. Test samples were placed 3 cm away and parallel to the right edge of the ASTM gel phantom and positioned parallel to the main magnetic field (B_0_) (Fig. 3a and c). Temperature measurements began with the probe positioned at the distal tip of the catheter, and then retracted along the catheter shaft during continuous scanning to sample all potential “hot spots” along the catheter shafts. Tests were performed during up to 20 min of scanning.

### In-vitro CMR imaging

CMR visibility performance of segmented and non-segmented braided catheter subassemblies was tested using a standard spin echo (SE) (TE/TR = 20/500 ms, flip angle = 50°, FOV = 300 mm, matrix = 192 × 192, slice thickness = 5 mm) and a GRE (TE/TR = 15/500 ms, flip angle = 50°, FOV = 300 mm, matrix = 192 × 192, slice thickness = 5 mm) sequence in a phantom per ASTM standard F2119–07 [23]. Tests samples were positioned 5 cm away from the phantom edges both parallel and perpendicular to the main magnetic field making a 90° curve to assess the effects of the device orientation in the magnetic field on the magnetic susceptibility artifact. A reference object with 25.4 mm diameter also placed in the phantom and the size of the magnetic susceptibility artifacts were evaluated according to ASTM standard F2119–07 (Fig. 3b).

### In-vivo CMR imaging and mechanical performance

Animal experiments received National Heart, Lung and Blood Institute (NHLBI) Animal Care and Use Committee approval and performed according to contemporary NIH standards in both Yorkshire and Yucatan swine. Segmented and non-segmented braided catheter samples were tested by experienced operators for expected in-vivo mechanical performance and CMR visibility during simulated pre-clinical right and left heart catheterization. To test in vivo mechanical performance, the aortic valve was traversed retrograde using both segmented and non-segmented-braided catheter samples from a femoral artery approach. In-vivo CMR conspicuity of segmented and non-segmented catheter subassemblies were tested using real-time bSSFP (TE/TR = 1.44/242.88 ms, flip angle = 80°, FOV = 350 mm, matrix = 144 × 160, slice thickness = 7 mm), while catheters were navigated into right heart chambers.

### In-vitro mechanical performance

**Fig. 4:**
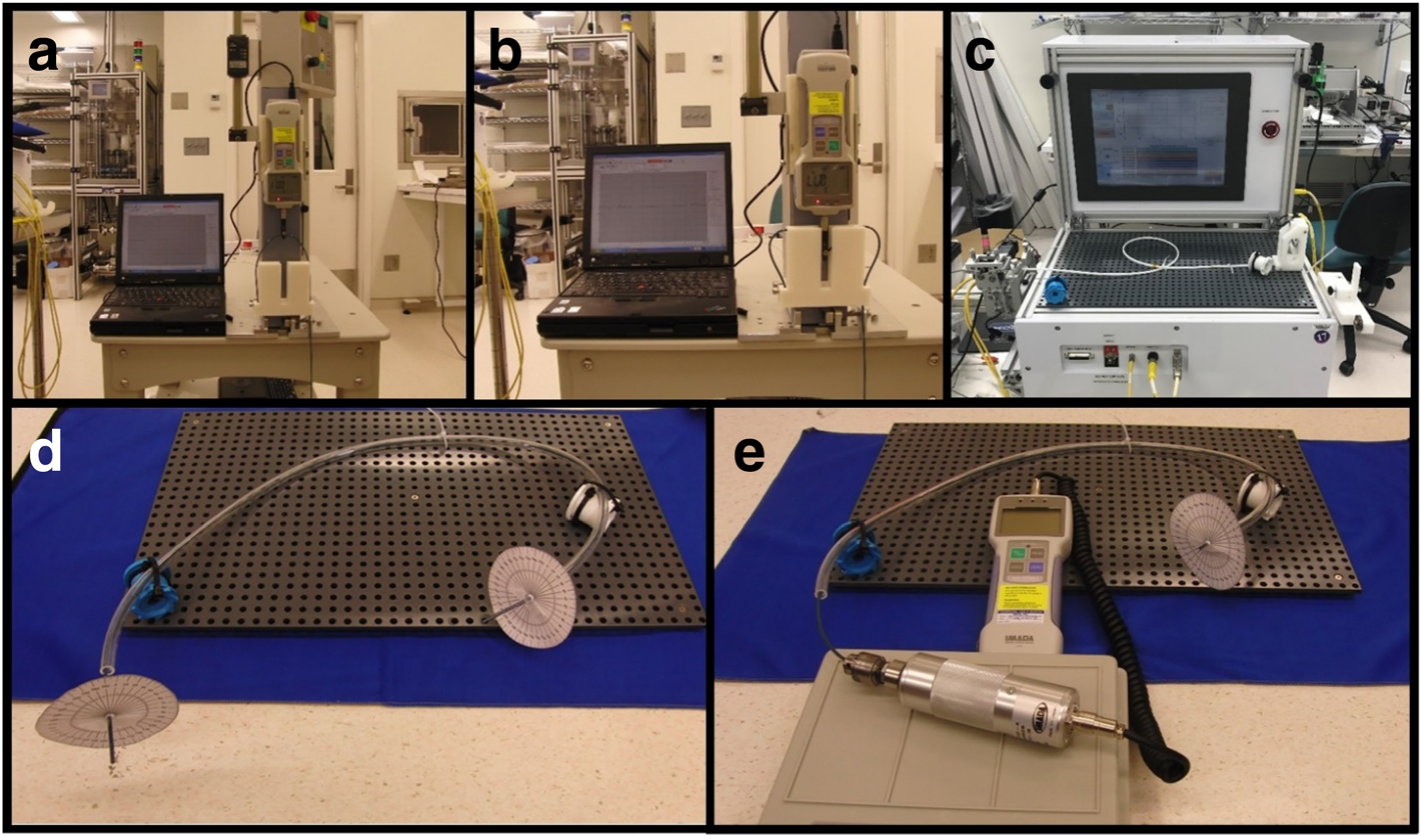
**a** and **b** Flexibility, **c** push-ability and **d** and **e** torque response test setups

Torque response, flexibility and push-ability characteristics of the segmented and non-segmented catheter prototypes and commercial comparators were tested to determine whether segmentation compromised mechanical integrity. The flexibility test was conducted using a digital force meter (IMADA Inc., Northbrook, Illinois, USA) attached to a programmable linear actuator while the catheter sample was attached to a 3-point bending apparatus (Fig. 4a). The bending properties of segmented and non-segmented braid samples and the commercial catheter were compared by pushing the samples for 15 cm and then allowing them to recoil to their initial state (Fig. 4b). The torque response test was conducted placing the catheter samples in an in vitro vessel phantom which mimicked the trajectory of a transfemoral catheter across the ascending aorta and connecting the proximal end to a digital torque meter (IMADA Inc.) (Fig. 4d and e). The torque response test measured the torque transmitted to the distal end from the proximal end (Fig. 4d), and the required torque to rotate the distal end 360 degrees (Fig. 4e). Transmitted rotation was tested 3 times per sample while the required torque for 360-degree rotation was tested 5 times per sample. The push-ability test was performed using a dedicated apparatus (IDTE 2000, MSI, Flagstaff, Arizona, USA) by pushing samples through a 55 cm vascular phantom (Fig. 4c). The load cell connected to the servo motor measured the reaction force during advancement 3 times per sample.

**Fig. 5:**
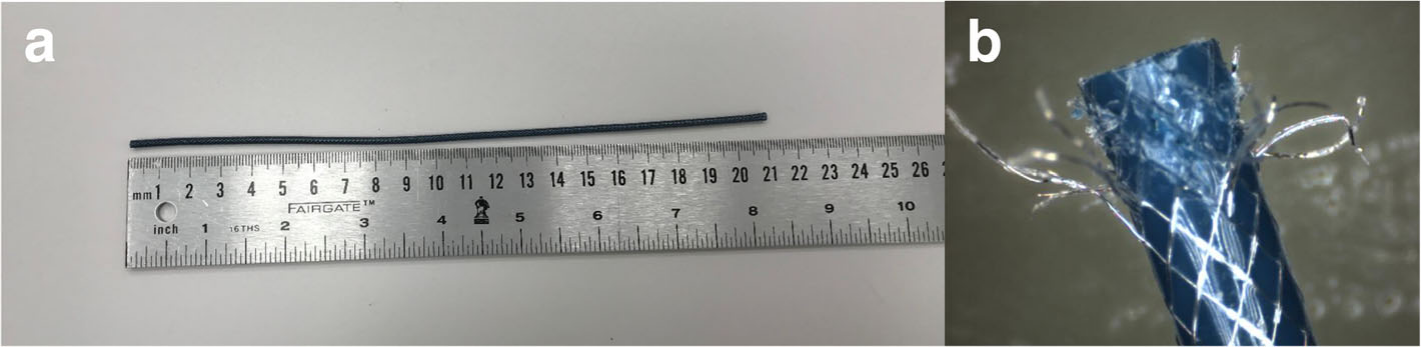
**a** 21 cm long dissected segmented braided catheter subassembly part and **b** detached and scraped braiding wires

First flexibility and then the push-ability tests were repeated 150 times on the segmented catheter subassembly to evaluate possible effects of a mechanically challenging interventional procedure on the insulation layer integrity. Torque response, flexibility and push-ability tests were repeated after mechanical stress tests. The 21 cm long flex-tested sample was dissected and tested for electrical insulation integrity (Fig. 5a). Extracted braiding was tested for conductivity (Fig. 5b). Using an electronic multimeter (Fluke 177, Washington, USA) and carefully tracing the individual braiding wires, each stress-tested braiding wire was assessed for a possible short circuit between wires.

## Results

### Prototype construction

**Fig. 6:**
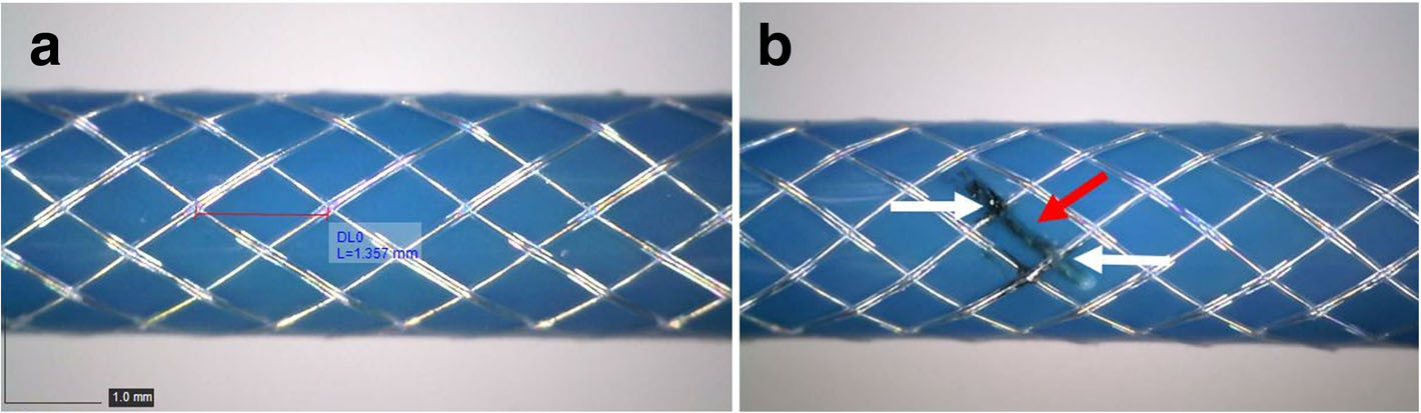
**a** Braiding uniformity and the node to node distance were confirmed under light microscope. **b** The segmented braid catheter constructed in-house. The segmentation points on the braiding layer are shown with white arrows (Blue insulation is discolored (red arrow) but not interrupted by the laser in-situ segmentation process)

Braiding uniformity was confirmed visually all along the catheter shaft for each sample and corroborated by measuring the consistency of node to node distance as 1.35 ± 0.01 mm (Fig. 6a) using a light microscope (Hirox KH-1300, Hirox Inc., Hackensack, New Jersey, USA). Laser, in-situ, interruption was also confirmed visually using the same setup (Fig. 6b).

### RF-induced heating

**Fig. 7:**
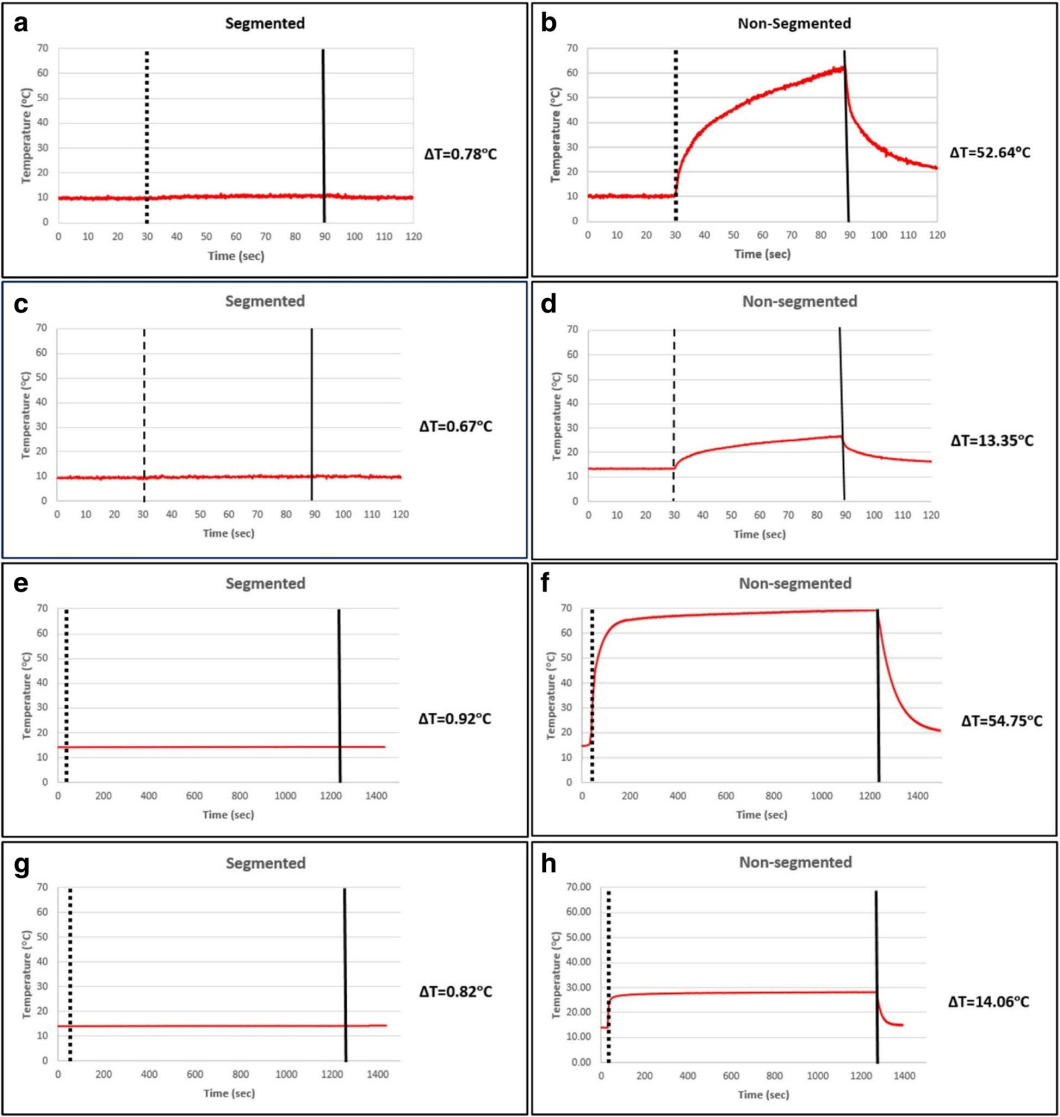
RF induced temperature rise of the segmented catheter subassembly after **a** 60 s bSSFP, **c** 60 s GRE, **e** 20 min bSSFP and **g** 20 min GRE scans. RF induced temperature rise of the non-segmented catheter subassembly after **b** 60 s bSSFP, **d** 60 s GRE, **f** 20 min bSSFP and **h** 20 min GRE scans

Segmentation greatly reduced heating under the test conditions (Fig. 7). Table 1 shows the temperature rise at the tip of the segmented- and continuous-braid catheters for bSSFP and GRE CMR scans.

**Table 1:** RF induced temperature rise at the distal tip

Sequence Type	Scan Time	Segmented Braiding (Δ°C)	Non-segmented Braiding (Δ°C)
Real-time bSSFP	60 s	0.78	52.6
Real-time bSSFP	20 min	0.92	54.8
Real-time GRE	60 s	0.67	13.4
Real-time GRE	20 min	0.82	14.1

### In-vitro CMR imaging

**Fig. 8:**
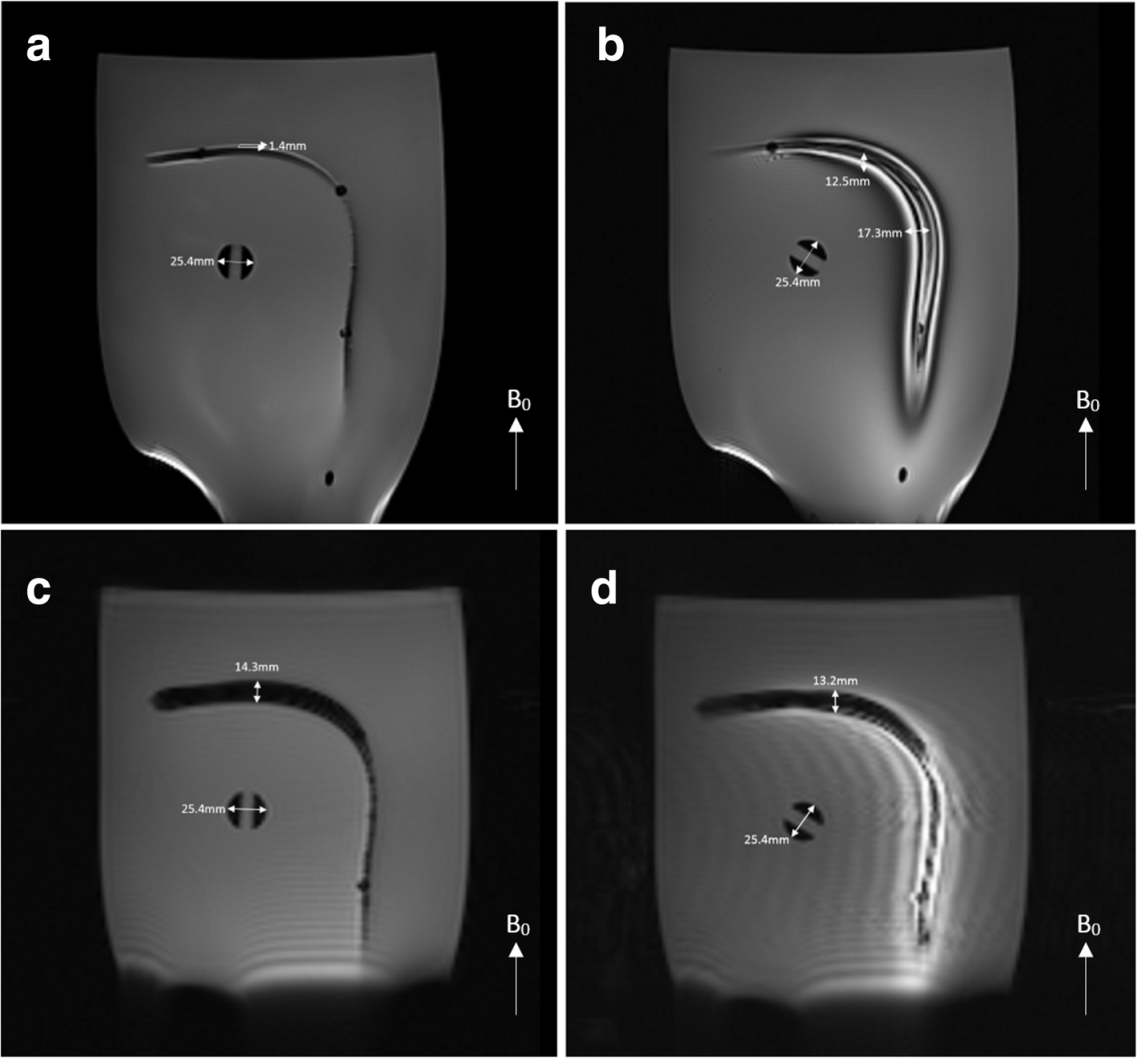
In-vitro CMR images and susceptibility artifact measurements. **a** Segmented catheter subassembly using SE and **c** GRE sequences and **b** non-segmented catheter subassembly using SE and **d** GRE sequences

Induced currents on the long conductive wires due to the E-field coupling during the RF excitation caused local flip angle amplifications which lead to artifacts in the close vicinity of the wire due to stimulated echoes. Electrically interrupting long conductive wires minimized the E-field coupling, thus reduced the local flip angle amplification (bright signal) but did not affect the magnetic susceptibility artifact (signal void). Via spin echo imaging, artifact size as a bright signal was measured as 1.4 mm for segmented and 12.5 mm for non-segmented sample perpendicular to the B_0_ and 17.3 mm for non-segmented sample parallel to B_0_, on the coronal plane. Using the GRE sequence, when samples were parallel to B_0_, the non-segmented sample caused a large blooming artifact while segmented sample did not cause any artifact. Conversely, when the samples were positioned perpendicular to B_0_, the artifact size as a signal void was measured as 14.3 mm and 13.2 mm for the segmented and non-segmented samples, respectively (Fig. 8).

### In-vivo CMR imaging and mechanical performance

**Fig. 9:**
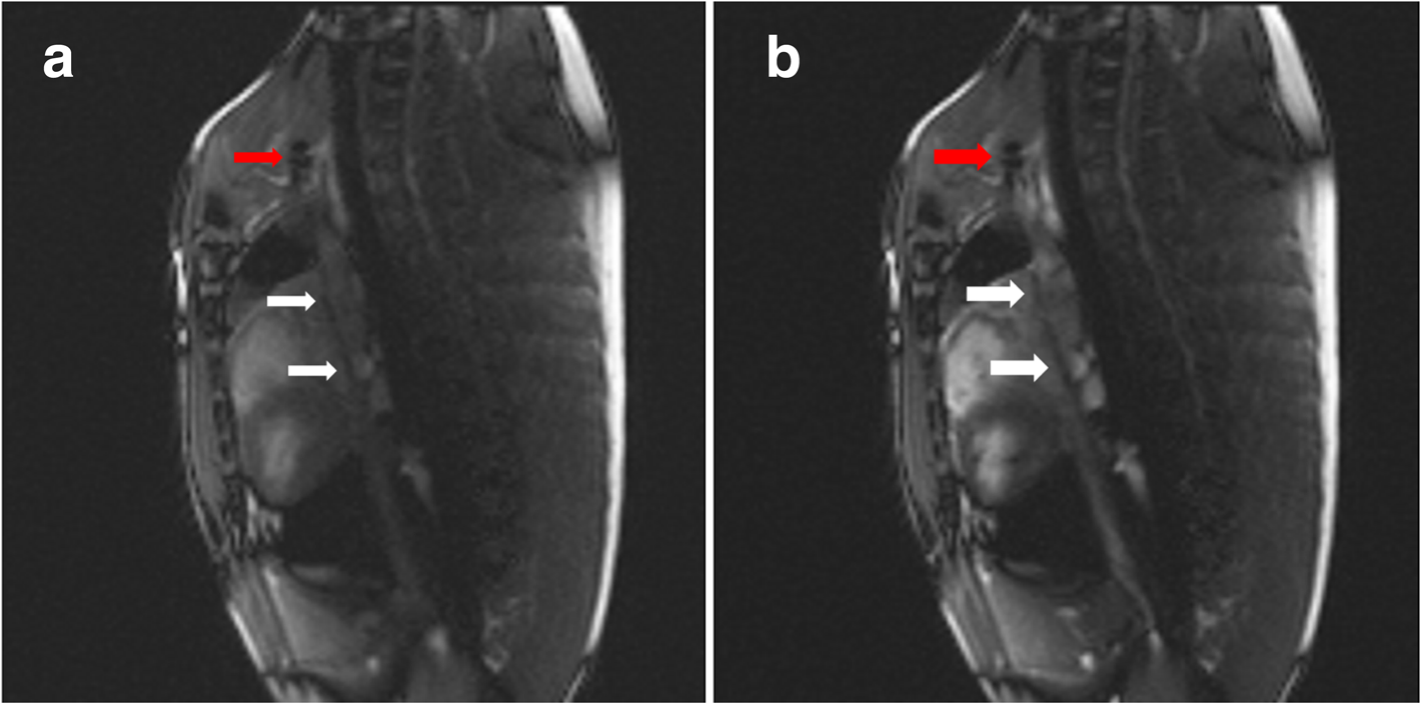
In-vivo CMR images. **a** Segmented braided and **b** non-segmented braided catheter subassemblies were advanced through the superior vena cava with a custom, iron-oxide coated guidewire in tandem during real-time bSSFP CMR (Red arrows indicate the tip of the custom, iron-oxide coated guidewire prototype, white arrows indicate the segmented and non-segmented catheter subassemblies)

Both segmented and non-segmented braided catheter subassemblies were visible in-vivo with a negative contrast during right heart catheterization in the Yorkshire pig, using a real-time bSSFP sequence (Fig. 9). The physician operator accessed the left ventricle retrograde across the aortic valve in under 10 s via transfemoral access using both segmented and non-segmented catheters. Both outperformed non-braided catheters and otherwise resembled commercial braided catheters in mechanical behavior.

### In-vitro mechanical performance

**Fig. 10:**
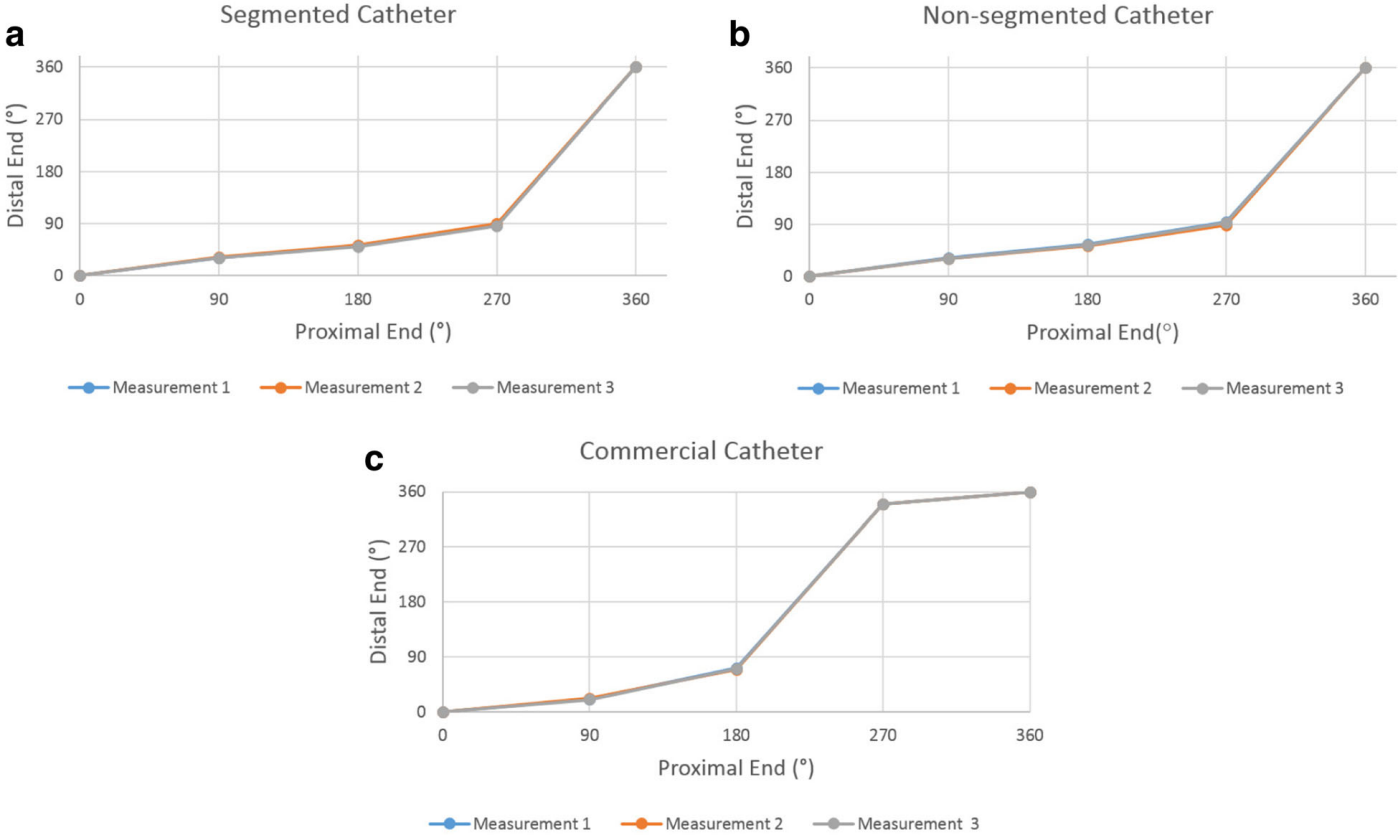
Torque transmission test results of (**a**) segmented, (**b**) non-segmented braid and (**c**) commercial catheters

Segmentation did not significantly alter torque-response. Both segmented and unsegmented catheters transmitted initial applied torque poorly from the proximal end to the distal end until 270-degree rotation, as is typical of commercial clinical devices. However, after the 270-degree rotation, sufficient baseline torque accumulated along the shaft to transmit torque effectively. Figure 10 shows the torque transmission test results for segmented and non-segmented braid catheters and the commercial catheter after 3 repetitions. Table 2 shows the required torque values for a 360-degree rotation of the distal tip.

**Table 2:** Torque requirement for a 360-degree rotation

Measurement #	Segmented catheter (kgf.cm^a^)	Non- Segmented catheter (kgf.cm^a^)	Commercial Catheter (kgf.cm^a^)
1	1.03	1.14	1.33
2	1.15	1.09	1.31
3	1.10	0.92	1.34
4	1.01	1.15	1.23
5	0.95	0.86	1.34
Mean	1.05 ± 0.07	1.03 ± 0.12	1.31 ± 0.04

^a^kgf.cm: Kilogram force per centimeter

**Fig. 11:**
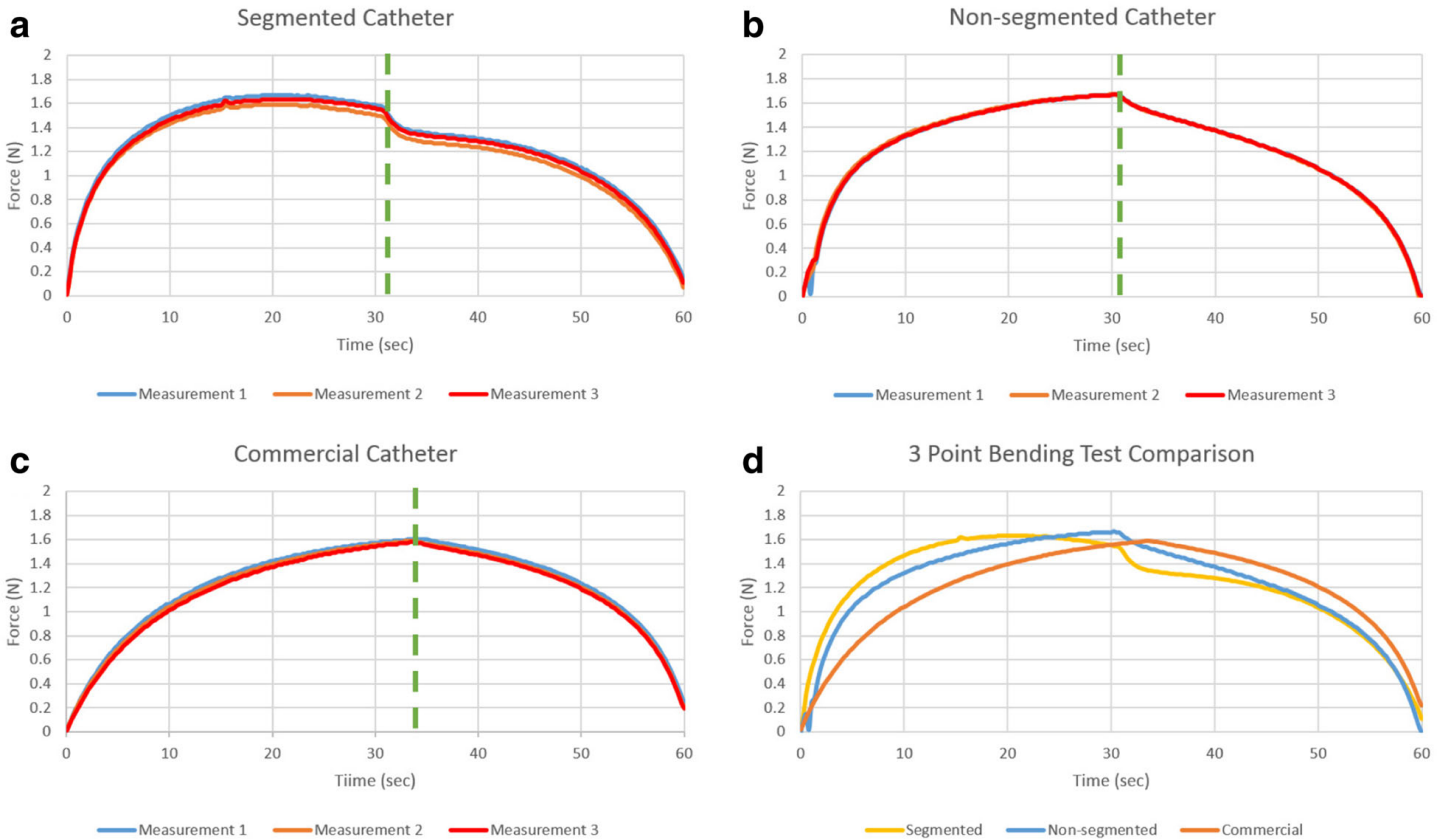
Bending stiffness test result of **a** segmented, **b** non-segmented braided and **c** commercial catheters and **d** the test results averages

Figure 11 shows the bending stiffness of all catheters after the 3-point bending test was performed 3 times for each sample. Kink resistance of segmented catheter shafts are comparable to non-segmented and commercial catheter shafts.

**Fig. 12:**
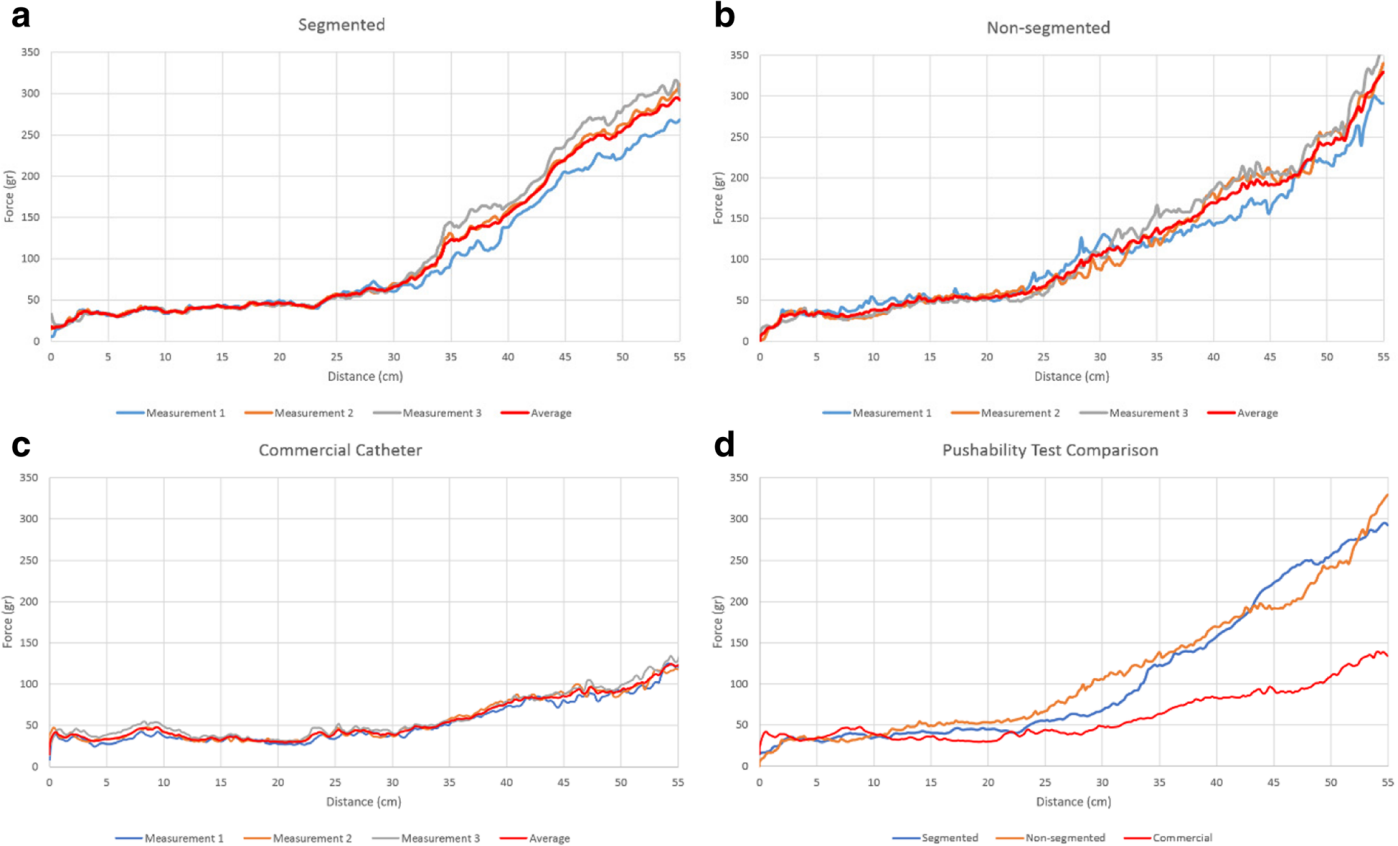
Push-ability test results of **a** the segmented catheter sample, **b** the non-segmented catheter sample and **c** the commercial catheter. **d** Comparison of all catheter samples (averaging test results)

Figure 12 shows the continuously measured push forces throughout the trajectory after three prior tests per sample. The performance of the segmented samples was comparable to non-segmented samples while that of commercial catheter was better than in-house developed prototypes.

**Fig. 13:**
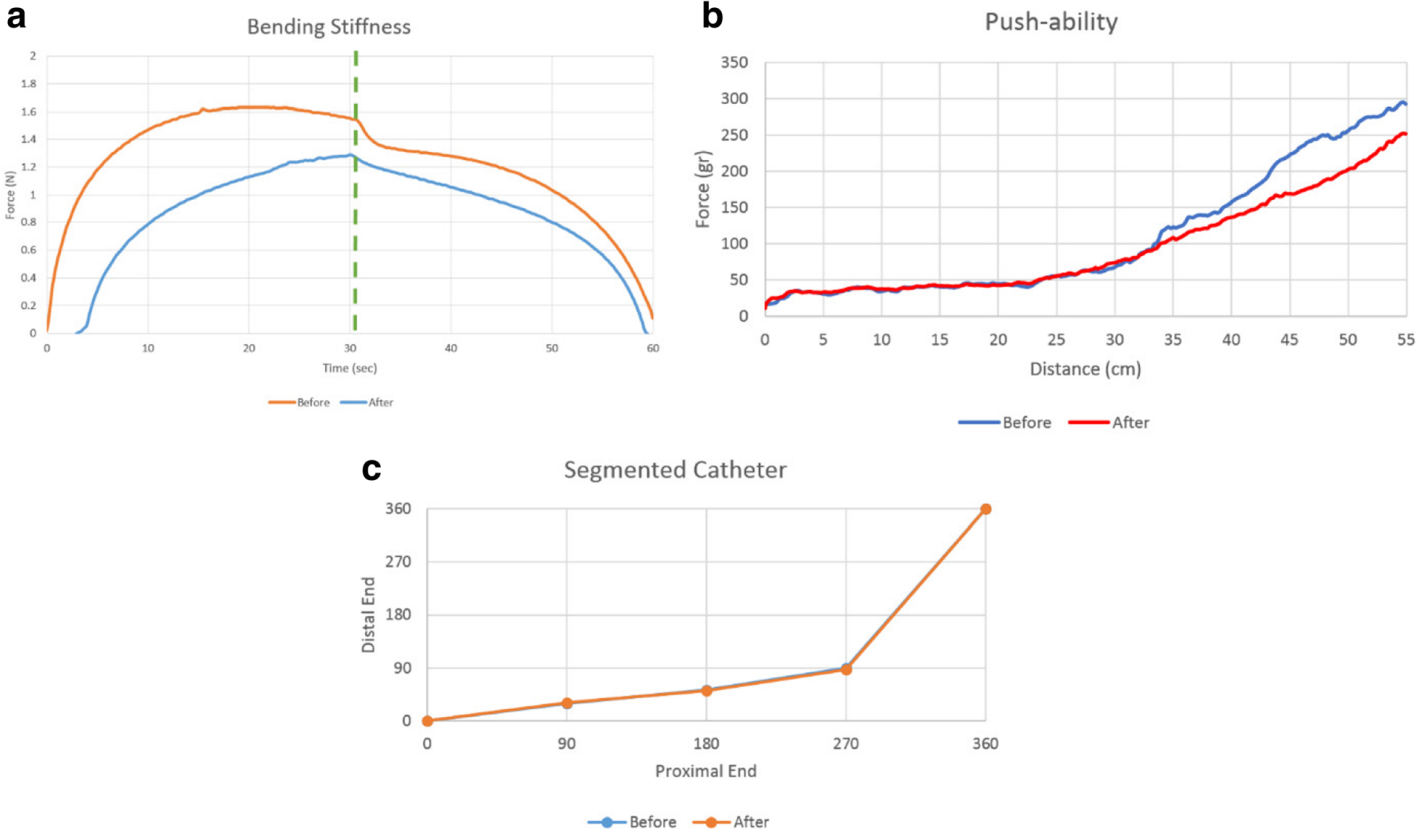
**a** Bending stiffness, **b** push-ability and **c** torque response test results of the segmented catheter after the deteriorating mechanical test

Figure 13 shows the flexibility, push-ability and torque response test results before and after the mechanically challenging test. Mechanical performance of the segmented catheter subassembly after the repeating mechanical tests was slightly degraded while electrical insulation remained intact and no short circuit was formed between braiding wires. The required torque for a 360-degree rotation was 1.51 ± 0.076 kgf.cm.

## Discussion

In this study, we show the feasibility of constructing CMR safe catheters that incorporate standard metal braiding into the shaft, a nearly ubiquitous design that imparts push-ability, torque response, and kink-resistance into catheters at low cost. A key feature of our design is that component stainless steel wires are individually insulated before braiding. After conventional braiding, the metallic fibers were strategically segmented, in-situ, at quarter-wavelength intervals to disrupt electrical continuity. At the same time, selected interruptions maintained catheter structural integrity and performance. We used transparent outer Pebax, which protected the inner polymer during laser cutting of the insulated wires. The segmentation pattern was selected to limit bidirectional rotation during laser cutting and assured braiding uniformity during manufacture. Benchtop mechanical parameters and catheter tracking in test phantoms were largely unaffected by the segmentation process and were comparable to commercial comparators.

Non-segmented samples demonstrated a CMR RF-induced temperature increase up to 54.8 °C. After segmenting the embedded braiding wires at quarter-wavelength intervals, the observed temperature increase was reduced to 0.92 °C, which is clinically insignificant [20].

The main limitation of our approach is that the braiding layers of conventional commercial braided catheters are typically formed using uninsulated wires. The proposed segmentation process requires component wires be insulated before braiding, to prevent wire crossing points from short-circuiting the otherwise electrically-isolated segments. However, the insulation layer thickness is negligible compared to the braiding wire diameter and imparts negligible mechanical change. The use of a polymer insulation layer does require that the process temperatures be kept under the melting temperature of the insulation polymer used. We have demonstrated that wire insulation is not disturbed by thermal reflowing at up to 190 °C to embed the braiding between two coaxial Pebax tubes since process temperature remains safely lower than 315 °C which is the melting point of the PFA polymer [24].

Both segmented and non-segmented braided catheter subassemblies were conspicuous under CMR in-vivo based on negative contrast. Moreover, segmenting the conductive braiding wires shorter than one-quarter wavelength at 1.5 T significantly reduced the artifact size caused by local flip angle amplification. Even though different steel alloys such as 316LVM would provide satisfactory susceptibility performance, despite segmentation, steel-braided catheters may still cause large magnetic susceptibility artifacts at 1.5 T [25], which may require specific materials selection, such as nitinol, titanium [26] or copper, to overcome. Considering the expected mechanical performance and complete CMR compatibility, an insulated nitinol wire would be the strongest candidate to replace stainless steel braiding wire [27].

## Conclusions

In conclusion, we eliminate RF-induced heating of standard conductive braided catheter designs, to be used in CMR catheterization procedures, by laser in-situ segmentation to shorten and electrically isolate braiding wires. We showed that this process does not alter the essential mechanical properties (push-ability, torque response and kink-resistance) of the braided catheters. This was achieved by constructing a braid structure using insulated metal wires, and then segmenting it in a strategical pattern in-situ, whereby electrical conduction is disrupted only 2 out of 16 wires at each segmentation location to eliminate RF heating. Therefore, the overall continuity of the braid structure is preserved to maintain mechanical integrity. Thanks to parametric programmability and repeatability of the process, this fabrication method can be applied to various length and gauge commercially available products using eqs. 1 and 2 and by replacing the bare metal wires with insulated non-magnetic ones during manufacture. This would be an attractive replacement for non-braided and/or non-metallic braided catheters which are inferior to their metallic counterparts.

## Abbreviations

ASTM: American Society for Testing and Materials
bSSFP: Balanced steady-state free precession
CMR: Cardiovascular magnetic resonance
Fr: French diameter (1Fr = 0.33 mm)
GRE: Gradient echo
ID: Inner Diameter
kgf.cm: Kilogram force per centimeter
LL: linear length
LV: Left ventricle/left ventricular
OD: Outer Diameter
PFA: Perfluoroalkoxyalkane
PPI: Pitches per inch
PTFE: Polytetrafluoroethylene
RF: Radiofrequency
rpm: Rotations per minute
